# Vaccines in Mixed Pulmonary Infection*Read before the third semi-annual meeting of the Southwest Texas District Medical Society at Corpus Christi, Texas, September 14, 1915.

**Published:** 1916-06

**Authors:** Theo. Y. Hull

**Affiliations:** San Antonio, Texas


### Vaccines in Mixed Pulmonary Infection.*

BY THEO. Y. HULL, M. D., SAN ANTONIO, TEXAS.

In presenting this preliminary report on the use of vaccines in the treatment of mixed pulmonary infections, it is not my purpose to present a long series of cases successfully treated, but rather to discuss the principles involved, the conditions that

*Read before the third semi-annual meeting of the Southwest Texas District Medical Society at Corpus Christi, Texas, September 14, 1915.

indicate or contraindicate their use, and incidentally to report such cases as may illustrate both good and bad results.

The idea of fighting bacteria with bacteria is not entirely new. As long ago as 1885 Cantani advocated the spraying into the lungs of cultures of putrefactive organisms, and Portucales in 1901 inoculated with syphilis to cure tuberculosis. The micro-organisms of erysipelas (Solles, 1893), the colon bacillus (Merreby, 1897) and yeast (Toürnier, 1900) have each been supposed to exert a healing influence upon tuberculosis. Even the bacillus typhosus was supposed to exert some influence for good upon the tuberculous process. The employment of these bacteria was based upon the theory of the antagonism between bacteria, the more recent and virulent infection rendering the soil unfit for the growth of the tubercle bacillus. According to this conception the interaction of various bacteria upon each other a mixed infection should prove beneficial to the patient.

Our present conception, however, of the secondary pulmonary infections is exactly the reverse. We not only look upon every invasion of other organisms as seriously complicating the case, but expect that at some time during its course every case of pulmonary tuberculosis will be complicated by such invasion. A mixed infection simply means that the body is fighting two or more diseases at the same time, each sapping its strength and lowering the general resistance. Each invading organism derives its sustenance from the host and in return floods the body with toxic substances of its own metabolism. Each invader renders the host just that much less able to defend itself against the more serious infection. A streptococcus infection or an influenza bacillus infection engrafted on an old tubercular base may be and very often is the inciting cause of an active tubercular process. In fact the most frequent cause of relapses in pulmonary tuberculosis is the so-called "winter cold." Without these concomitant infections a majority of cases would go on uninterruptedly to recovery. Because of the susceptibility of the tuberculous invalid to such infection the Frenchman was more than half right when he said that the worst thing that can happen to a tuberculous person is to come in contact with a consumptive. The consumptive is almost invariably the carrier of pathogenic micro-organisms, other than the tubercle bacillus. More recent experiments have shown that the relationship between these invading organisms is symbiotic rather than antagonistic. From these experiments we have learned that the tubercle bacillus grows more luxuriantly in the presence of other bacteria. The true significance of a multiple invasion is seen in the common observation that a pure tuberculosis tends to heal while a mixed infection tends to become intractable.

In combating the inroads of the tubercle bacillus—tuberculin—a tubercle vaccine has long been used, but it has no influence upon the other organisms that may be present. To meet this condition of multiple infection, other vaccines have been employed. The use of any vaccine rests upon the biological law that whenever the body is invaded by a foreign protein (parasite) the host strives to elaborate a special ferment by which the invader may be destroyed. The certainty and the rapidity with which this may be done depends upon the physical condition of the host; that is, the amount of ferment it may elaborate. In every case of mixed infection the question arises: can we stimulate the production of the specific ferment in sufficient quantities to be of value? The answer to this question must be found chiefly in the physical condition of the patient and the discovery of the offending organism or organisms. It may be that the patient is so depleted that the body cannot respond to the specific stimulation. Possibly the following case may illustrate this point more clearly:

Case 1.—Miss S., age 24 years, 5 feet 8 inches in height, weight 133 pounds, gave a history of a persistent cough for the past eight years, expectoration for five years, sputum streaked with blood at times, and frequent pleuritic pains. She appeared for examination at 4 p. m. Her blood pressure was 122 systolic, and 70 diastolic, temperature 99.4, pulse 96, respiration 24, and *hemoglobin 15.* The physical examination indicated an old fibroid phthisis involving both lungs, upon which had been engrafted a general influenza bacillus infection. At the time she has received about twenty doses of an autogenous vaccine without any apparent effect. The remarkable feature of this case was the low percentage of hemoglobin. The point that I wish to call your attention to is that in such a condition of the blood we could not expect any response in the production of specific ferments. It was simply impossible for the body to respond to any protein stimulation. The cutaneous tuberculin test was negative for the same reason. In my opinion the use of any vaccine was contraindicated by the low hemoglobin content of the blood, if none other.

The second point to be observed, the discovery of the offending organism or organisms, is also of the first importance. The failure to do so always means failure in results. Notwithstanding the fact that in every case of pulmonary tuberculosis there is always the possibility that other bacteria may be at work, we must not forget that the invading organisms may not be the cause of the symptoms presenting. The following case will illustrate this point:

Case 2.—Mrs. K., age 30 years, height 5 feet 6 inches and weight 114 pounds. Her husband died of a rapid pulmonary tuberculosis ten months before. At the time of the examination, 11 a. m., her blood pressure was 105 systolic and 60 diastolic, temperature 102.8, pulse 120, respiration 32, hemoglobin 45. There was a general infection involving the entire left lung and the upper lobe of the right. There was a distinct valvular murmur over the left apex. She had had several hemorrhages. The sputum contained numerous tubercle bacilli and a few other bacteria. The urine was slightly albuminous. She also had received some twenty or more doses of an autogenous vaccine with no results. There were several reasons for the failure of the vaccine in this case. Besides the low hemoglobin content of the blood, there was a general and severe tubercle bacillus infection. The other organisms present were incidental and not causative factors in the symptom-complex and consequently no vaccine containing these organisms could have any beneficial result. I do not think we can emphasize this point too much. The injection of large numbers of dead micro-organisms is not only useless but may be distinctly harmful.

A third condition that I wish to call your attention to is that of an anaphylaxis. We may conceive the protein molecule to be made up of several layers surrounding an innermost poison group. If by any means the digestion of this molecule proceeds too far and the poison group is liberated characteristic symptoms may appear and a fatal termination may ensue. In the following case the increased activity of the tubercular infection may be attributed to the general weakening of the patient's power of defense:

Case 3.—Mr. M., age 27, height 5 feet 9 inches, weight 140 pounds. He was taken sick January, 1913, with la grippe. The attack was followed by a cough, expectoration and fever. The sputum became blood streaked about April, but no free hemorrhage occurred. There was also a history of an attack of syphilis several years before the onset of the tuberculosis. At the time of the examination, 3 p. m., October, 1913, the patient's bloodpressure was 124 systolic and 85 diastolic, temperature 100, pulse 120, respiration 28, and hemoglobin 70. The sputum contained many tubercle bacilli, streptococci and micrococci of catarrh. The urine was normal. The physical examination of the chest showed the apices of both lungs to be involved and the left ventricle of the heart to be enlarged. This was a second-stage case, active. Rest in bed and careful dieting failing to reduce the temperature or lessen the blood-streaked expectoration, an autogenous vaccine was prepared containing streptococci and micrococci of catarrh and administered at the usual five-day period. The first of four doses produced no effect either upon the temperature or upon the character of the expectoration. Following the fifth dose, however, a severe reaction occurred. The temperature rose to 104 and the cough and the expectoration increased. The tubercular process became more active and persisted until death intervened a few months later. There was every appearance of anaphylaxis. The individual power of resistance to the progress of the tubercle bacillus seemed to have been abolished. What part the syphilitic infection played in producing this condition is problematic. The acute exacerbation was undoubtedly due to the sudden liberation of an undue proportion of the poison group in the protein molecule following an overdose of the vaccine.

In some of these cases we sometimes meet a fourth condition, due to the depressing effect upon the heart of the sudden liberation of the protein poisons.

Case 4.—Mr. T., age 40, height 5 feet 8 inches and weight 175 pounds. He had suffered three or four attacks of asthma, and he complained of much shortness of breath. A first cold was contracted in June, followed by cough and expectoration. A second and more severe cold was contracted September 1, 1914, followed by a continued fever, cough and expectoration. The daily range of temperature was typical of tuberculosis. He appeared for examination on October 12th at 3 p. m. His blood-pressure was 125 systolic and 80 diastolic, temperature 99, pulse 100, respiration 23, and hemoglobin 85. The physical examination revealed the evidence of old tubercular lesions. The sputum contained many pneumococci but no tubercle bacilli. It was evident that we were dealing with a pneumococcus bronchitis engrafted upon a tubercular base with grave danger of lighting up the old tubercular processes. A pure pneumococcus vaccine was prepared and the first dose of 50 million administered. For three days preceding this the temperature rose to 100.8 each afternoon. The day following the temperature rose to 101.4 at 10 a. m. On the fifth day a second dose of 100 million pneumococci was given. After this the temperature began to decline. Four days later a third dose of 125 million was administered at 12:30 p. m. The next day the temperature dropped to normal, and remained so during the rest of my observation. The point in this case to which I wish to call your attention was the disturbance of the cardiac function for several hours following the third dose. The patient was greatly frightened and the nurse reported that he became cold and damp and the pulse very weak about twelve hours after the injection. The disease had terminated by crisis. Six weeks later the patient's blood pressure was 120 systolic and 90 diastolic, temperature 98.6, pulse 88, and hemoglobin 93. The cough and expectoration had ceased. The latent tubercular processes were evidently undisturbed.

The following cases illustrate several classes in which good may be expected from carefully given vaccines.

Case 5.—Miss C., age 17, weight 86 pounds. The history of this case showed an attack of la grippe (so-called) January, 1911, followed by cough, fever and expectoration, a hemorrhage and an attack of pleurisy later. On March 21, 1913, at 4 p. m., the patient appeared for examination. Her blood pressure was 92 systolic and 60 diastolic, temperature 100, pulse 120, respiration 26, and hemoglobin 55. The sputum contained many tubercle bacilli, streptococci, staphylococci, and micrococci catarrhalis. Physical examination of the chest showed both apices to be involved. The diagnosis was a second stage-case, active, complicated by severe mixed infection. A stock vaccine was administered in four graduated doses at intervals of five days. The daily range of temperature was before the first dose 97.4, a. m., to 101.4, p. m. From the first to the fourth dose it ranged somewhat higher, 97.2, a. m., to 102, p. m. The day following the fourth dose the temperature dropped to normal and remained so for several weeks. Later examination of the sputum showed the absence of the streptococci, staphylococci and the micrococci of catarrh but the tubercle bacilli persisted. Since rest and diet failed to reduce the temperature prior to the use of the vaccine, it is plainly evident that the mixed infection was responsible for many of the symptoms. The patient considered herself well, adopted Christian Science, and died December, 1914.

Case 6.—Mrs. A. This patient was treated for pulmonary tuberculosis in 1911 with tuberculin. The disease was arrested and the weight increased from 81 to 108 pounds. In January, 1915, she contracted a severe cold, followed by cough, expectoration and fever. There was grave danger of lighting up the old processes. The sputum contained numerous pneumococci and micrococci of catarrh. A vaccine was made from these two organisms. Nine doses were administered at intervals of five days. The initial dose contained 50 million of each organism and the last dose 150 million of each. The outcome was the relief of the most distressing symptoms.

Case 7.—Miss H., age 43. She had suffered from frequent colds and from bronchitis for the past two years. On examination her blood-pressure was 126 systolic, 84 diastolic, temperature 98.4, pulse 70, respiration 24, and hemoglobin 80. The physical signs indicated an old case of fibroid phthisis. There was also a pronounced cardiac murmur (mitral). The sputum contained staphylococci, streptococci, and pneumococci, the staphylococci predominating. A vaccine, autogenous, containing these organisms was administered, nine doses in all being given. The first dose contained 200 million staphylococci and 10 million each of the streptococci and pneumococci. Great improvement in the chief symptoms followed. There was no lighting up of the old processes, and the patient subsequently gained several pounds in weight.

Case 8.—Mrs. M., age 24, height 5 feet 5 inches and weight 140 pounds. This case represents a type quite frequently seen. The onset of the disease was July, 1913; several hemorrhages and the usual symptoms of cough, fever and expectoration. The examination was made on November 26, 1914, at which time the blood pressure was 116 systolic and 75 diastolic, temperature 99.2, pulse 100, respiration 36, and hemoglobin 90. Both apices were involved. The sputum contained streptococci, micrococci of catarrh and tubercle bacilli. The diagnosis, second stage, active. On December 8, 1914, the first vaccine of 50 million streptococci and 50 million micrococci of catarrh was administered. The fourteenth dose of 500 million each was given March 4, 1915. At the beginning of the treatment the temperature ranged from 98, a. m., to 100.4, p. m.; at the close 97, a. m., to 99, p. m. On April 10th, at 2:30 p. m., the blood-pressure was 106 systolic and 85 diastolic, temperature 98.8, pulse 84, respiration 28, and hemoglobin 95. The subsequent history of the patient was uneventful.

In the foregoing cases I have sought neither to praise unduly nor to withhold praise where it is due, but to state conditions which we meet and to draw what conclusions we may therefrom. Vaccine therapy is a part of our armamentarium in fighting tuberculosis. It represents one of the greatest advances, next to the discovery of bacteria, in scientific medicine, but like many other valuable agents vaccines may be misused. In their use, accuracy of diagnosis is necessary to success. Bacteria are specific proteins in this that their presence in the host stimulates the production of specific ferments (antibodies). The vaccine used in combating any bacterial infection must contain the identical or very closely related protein as the invading organism. In the second case presented over twenty doses of an autogenous vaccine had been given without the slightest advantage because the bacteria used in making the vaccine were not identical or even related to the bacterium causing the distressing symptoms.

But it is not enough to be certain of the offending organism, the diagnosis must determine whether we are dealing with a general or a local infection. As a rule localized infections respond favorably to the proper vaccines while general infections do not respond at all. When a vaccine is introduced into the blood it is soon distributed throughout the tissues of the body and there stimulates the specific ferments. In strictly localized and circumscribed infections this is possible, but in cases of a general infection it is impossible, for the power of the tissues to respond to the stimulus is exhausted. Cases Nos. 1 and 8 illustrate this point. No. 8 was a localized infection, and the body responded to the stimulation. The same was true of cases Nos. 5, 6 and 7. No. 1, on the other hand, the general infection precluded the possibility of producing a greater supply of defensive ferments.

In the use of vaccines we must not forget that there are cases in which vaccines do positive injury. We know that every protein molecule contains a poison group. We have no way at present to eliminate this poison group in our vaccines. This is also one of the most difficult conditions to diagnose correctly. Cases Nos. 3 and 4 show what may happen with the too sudden liberation of this protein poison. Upon these two points depends the future of vaccine therapy. Until our laboratories can prepare a vaccine that may be used effectively and yet without danger the diagnostician must play an exceedingly important part and the use of the vaccines must be confined to the more expert.

If a vaccine can be prepared from which the poison group of the protein molecule is eliminated or rendered innocuous, the general and indiscriminate use of such agents may be permissible; but in the present state of our knowledge and our inability to render them harmless, bacterial vaccines of all kinds should be used in the treatment of pulmonary infections with caution. The words vaccine and panacea are not synonymous.

